# BreastDefend™ prevents breast-to-lung cancer metastases in an orthotopic animal model of triple-negative human breast cancer

**DOI:** 10.3892/or.2012.1936

**Published:** 2012-07-26

**Authors:** JIAHUA JIANG, ANITA THYAGARAJAN-SAHU, JAGADISH LOGANATHAN, ISAAC ELIAZ, COLIN TERRY, GEORGE E. SANDUSKY, DANIEL SLIVA

**Affiliations:** 1 Cancer Research Laboratory, Methodist Research Institute, Indiana University Health, Indianapolis, IN 46202; 2 Amitabha Medical Clinic and Healing Center, Sebastopol, CA; 3 Department of Pathology, Indiana University School of Medicine, Indianapolis, IN, USA; 4 Department of Medicine, Indiana University School of Medicine, Indianapolis, IN, USA; 5 Indiana University Simon Cancer Center, Indiana University School of Medicine, Indianapolis, IN, USA

**Keywords:** triple negative breast cancer cells, lung cancer metastasis, dietary supplement, urokinase plasminogen activator, C-X-C chemokine receptor-4

## Abstract

We have recently demonstrated that a natural dietary supplement BreastDefend (BD), which contains extracts from medicinal mushrooms (*Coriolus versicolor, Ganoderma lucidum, Phellinus linteus*), medicinal herbs (S*cutellaria barbata, Astragalus membranaceus, Curcuma longa*), and purified biologically active nutritional compounds (diindolylmethane and quercetin), inhibits proliferation and metastatic behavior of MDA-MB-231 invasive human breast cancer cells *in vitro*. In the present study, we evaluated whether BD suppresses growth and breast-to lung cancer metastasis in an orthotopic model of human breast cancer cells implanted in mice. Oral application of BD (100 mg/kg of body weight for 4 weeks) by intragastric gavage did not affect body weight or activity of liver enzymes and did not show any sign of toxicity in liver, spleen, kidney, lung and heart tissues in mice. Moreover, BD significantly decreased the change in tumor volume over time compared to the control group (p=0.002). BD treatment also markedly decreased the incidence of breast-to-lung cancer metastasis from 67% (control) to 20% (BD) (p<0.05) and the number of metastases from 2.8 (0.0, 48.0) in the control group to 0.0 (0.0, 14.2) in the BD treatment group (p<0.05). Finally, anti-metastatic activity of BD *in vivo* was further confirmed by the downregulation of expression of *PLAU* (urokinase plasminogen activator, uPA) and *CXCR4* (C-X-C chemokine receptor-4) genes in breast tumors. In conclusion, BD may be considered as a biological therapeutic agent against invasive breast cancers.

## Introduction

The majority of newly diagnosed cancers are breast cancers and breast cancer is also a leading cause of cancer death in women globally (1). In spite of the early diagnosis, radiation and chemotherapy, breast cancer is the second leading cause of cancer death in the United States (2). One of the major reasons for such a high morbidity and mortality of breast cancer is the invasive behavior of breast cancer cells leading to cancer metastasis. Certain natural/dietary compounds, presented in vegetables and fruits, can affect various signaling pathways and molecular targets leading to their possible use in the combination therapy (3). The use of dietary supplements is highest among breast cancer survivors (4) suggesting that these supplements can prevent the exacerbation of comorbid conditions associated with breast cancer (5). Proper evaluation of toxicity and biological effects of polybotanical dietary supplements in cancer in general and in breast cancer in particular is of great importance.

BreastDefend™ (BD) is a polybotanical dietary supplement which inhibits growth and invasive behavior of highly metastatic human breast cancer cells *in vitro*(6). BD contains mycelium from Asian medicinal mushrooms (*Coriolus versicolor, Ganoderma lucidum, and Phellinus linteus*), which separately suppressed growth and inhibited invasiveness of breast cancer cells by different mechanisms (6–10). In addition, some of the natural agents in BD also demonstrated anticancer activities against breast cancer cells. For example, extracts or purified compounds from *Scutellaria barbata, Astragalus membranaceus* and *Curcuma longa* suppressed growth, induced oxidative stress and apoptosis, inhibited breast cancer cell invasiveness and prevented breast cancer metastases in mice (11–14). Quercetin, a flavonoid found in fruits, vegetables, leaves and grains inhibited proliferation of invasive breast cancer cells and its combination with other polyphenols further suppressed tumor growth and site-specific metastasis (15,16). Finally, 3,3′-diindolylmethane (DIM), the biologically active compound derived from the digestion of indole-3-carbinol, found in cruciferous vegetables such as broccoli, Brussels sprouts, cabbage and kale suppressed growth, migration and invasion of metastatic breast cancer cells (17,18).

In the present study, we evaluated toxicity and anti-cancer activities of BD in an animal model of breast-to-lung cancer metastasis with triple-negative highly invasive humane breast cancer cells MDA-MB-231 implanted in the mammary pad of nude mice. Here, we show that BD is not toxic and its oral application significantly suppresses time-dependent increase in tumor sizes and inhibits breast-to-lung cancer metastasis. In addition, BD inhibits expression of pro-metastastic genes *PLAU* and *CXCR4*, in breast cancer xenografts. Our results confirm that BD is not toxic and inhibits growth and metastasis of invasive human breast cancer cells *in vivo*.

## Materials and methods

### Cell culture and reagents

Highly invasive human breast cancer cells (MDA-MB-231) were obtained from ATCC (Manassas, VA). MDA-MB-231 cells were maintained in DMEM medium supplemented with penicillin (50 U/ml), streptomycin (50 U/ml), and 10% fetal bovine serum (FBS). Media and supplements came from Gibco BRL (Grand Island, NY). FBS was obtained from Hyclone (Logan, UT). BreastDefend (BD) was supplied by EcoNugenics^®^, Inc. (Santa Rosa, CA). BD contains the following active weight components: herb and active nutritional blend 57.56%, [Quercetin (98% bioflavonoids), Turmeric rhizome extract (*Curcuma longa*) complex with enhanced bioavailability (BCM-95^®^), *Scutellaria barbata* herb extract, and *Astragalus membranaceus* root extract], mushroom mycelium blend 30.3% (*Coriolus versicolor, Ganoderma lucidum, Phellinus linteus*), and 3,3′-diindolylmethane 12.12%. BD is manufactured consistent with the FDA Good Manufacturing Practices (GMP) regulation for dietary supplements as defined in 21 CFR§111, for batch to batch consistency and quality controls. BD stock solution was prepared by dissolving BD in dimethylsulfoxide (DMSO) at a concentration 25 mg/ml and stored at 40°C.

### Toxicology studies

Toxicity of BD was evaluated in the 6-week old female nude mice (Harlan, Indianapolis, IN, USA). The mice were acclimatized for 1 week, and BD was applied by intragastrical gavage 5 times/week for additional 4 weeks at the following doses: 0, 100, 200 and 400 mg/kg of body weight, n=10 per group. The body weight was evaluated three times per week. At the end of the experiment animals were euthanized by CO_2_ inhalation. Blood was collected and a gross pathology examination performed. Liver, spleen, kidney, lung and heart were harvested, fixed in 10% neutral buffered formalin at 4°C for 24 h followed tissue processing overnight, and then embedded in paraffin. Five-micrometer sections were stained with hematoxilyn and eosin (H&E). The levels of alanine aminotransferase (ALT), aspartate aminotransferase (AST), alkaline phosphatase (ALP), albumin and total protein were determined at the Department of Pathology and Laboratory Medicine, Indiana University (Indianapolis, IN, USA).

### Human breast tumor xenograft experiments

MDA-MB-231 cells (1×10^6^) in 0.2 ml DMEM were implanted subcutaneously in the mammary fat pad of the 6-week old female nude mice (Harlan). After 1–2 weeks of implantation with tumor cells, when tumors reached ~20–30 mm^3^, the animals were randomized into control and treatment groups (15 animals per group). The animals received intragastrical gavage 5 times/week with water (control) or 100 mg BD/kg of body weight (treatment) for additional 33 days. The tumor size was measured using calipers, and the tumor volume was estimated by the formula: tumor volume (mm^3^) = (W × L) 2 × 1/2, where L is the length and W is the width of the tumor. At the end of the experiment (day 33), the tumors were snap frozen and stored separately in liquid nitrogen. The lung were harvested, fixed in 10% neutral buffered formalin at 4°C for 24 h followed tissue processing overnight, and then embedded in paraffin. Five-micrometer sections were stained with hematoxylin and eosin (H&E) and number of metastasis counted under the light microscope.

The protocol for animal experiments was approved by the Animal Research Committee at the Methodist Hospital according to the NIH guidelines for the Care and Use of Laboratory Animals.

### Quantitative RT-PCR

The quantitative real-time polymerase chain reaction (qRT-PCR) was performed using the ABI PRISM 7900HT Fast Real-Time PCR system (Applied Biosystems) according to the manufacturer’s instructions. Total RNA was isolated from tumors with RNAeasy (Qiagen, Valencia, CA). The RNA samples were reverse transcribed into cDNA (RT-PCR) using random hexamer primers and TaqMan reverse transcription kit (Applied Biosystems). The cDNA (100 ng per sample) was subjected to qPCR analysis in quadruplicate using forward and reverse primers, TaqMan Universal Master Mix, and probe (10 μl per reaction) in fast optical 96-well plates. The data were analyzed using the ABI PRISM 7900 relative quantification (DDCt) study software (Applied Biosystems). In this study we have used primers for *PLAU, CXCR4, EZR, HRAS, S100A4, CDKN1A* and *HTATIP2* genes with *β-actin* gene as internal control (Applied Biosystems). The gene expressions levels are normalized to β-actin and are presented as arbitrary fold changes compared between control and treated groups.

### Statistical analysis

Toxicology analyses of plasma were summarized using median (min, max) and compared across groups using Kruskal-Wallis tests and Mann-Whitney U tests with significance level adjusted using the Bonferroni correction. The changes in body weight and tumor volume over time were tested using a random effects mixed model. Metastasis incidence was summarized using percentage of animals with metastases and compared between control and BD treatment groups using Fisher’s exact test. Metastasis multiplicity and qRT-PCR data were summarized using median (min, max) and compared between control and BD treatment groups using Wilcoxon rank sum test.

## Results

### Toxicity of BD in vivo

**Figure 1:**
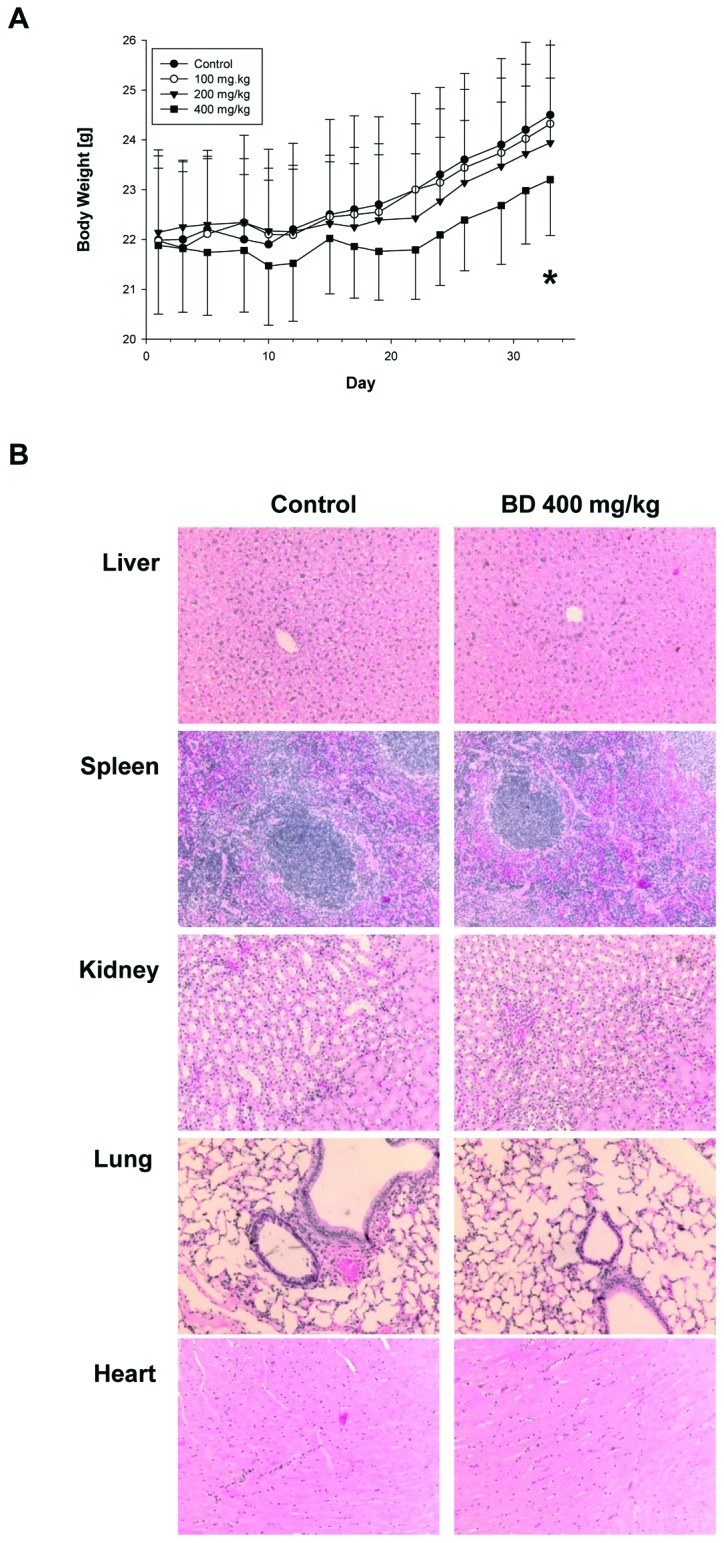
Toxicity of BD. Female nude mice were gavaged with BD (0, 100, 200 and 400 mg/kg of body weight, n=10 per group) 5 times/week for 4 weeks. (A) The body weight was evaluated three times per week. ^*^p<0.001 change in body weight overtime control vs BD 400 mg/kg. (B) Liver, spleen, kidney, lung and heart were harvested and stained with H&E as described in Materials and methods. The pictures are representative from control and high dose BD treatment (400 mg/kg) groups.

Our recent study demonstrated cytostatic effect of BD on human breast cancer cells MDA-MB-231 (6). Although BD was not toxic for these cells *in vitro*, systemic toxicity in animals needs to be evaluated. To evaluate the toxicity of BD *in vivo*, female nude mice were orally gavaged by 0, 100, 200 and 400 mg BD per kg of body weight for 33 days as described in Materials and methods. A seen in Fig. 1A, all groups demonstrated increase in body weight but the increase in 400 mg/kg group was decreased by 5% to control group and this change in body weight overtime was significant (p<0.001). In addition, gross necropsy did not show any sign of toxicity and weights of liver, spleen, kidney, lung and heart were not different between the treatment and control groups (not shown). H&E staining of control and highest BD dose treatment group (400 mg/kg) of liver, spleen, kidney, lung and heart also did not demonstrate any abnormalities (Fig. 1B). Although the liver enzyme profiles in plasma (ALT, AST and ALP) were not changed by BD treatment (0–400 mg/kg), the levels of albumin and total protein were decreased at the 200 and 400 mg BD per kg groups (Table I). Therefore, 100 mg BD/kg dose was decided to be used in our experiments.

### BD suppresses tumor growth and prevents breast-to-lung cancer metastasis in an orthotopic model of human breast cancer

**Figure 2:**
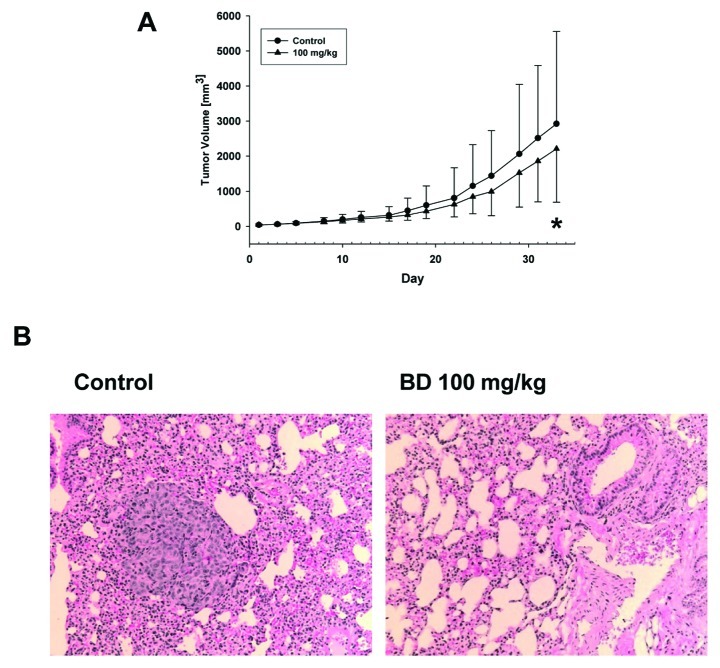
Effect of BD on the tumor size. MDA-MB-231 cells were injected into mammary fat pad into female nude mice and treated with water (control) or 100 mg BD/kg of body weight 5 times/week for daily for 33 days (n=15 mice per group) as described in Materials and methods. (A) Tumor volumes: data are the mean ± SD and the changes in tumor volumes were statistically evaluated by a random effects mixed model (p=0.002). (B) Breast tumors were induced as described above. At the end of experiments, mice were sacrificed, lungs harvested and stained with H&E as described in Materials and methods. Representative picture of control and BD treatment are shown. The data analysis is in Table II.

Based on our data demonstrating that 100 mg/kg of BD is not toxic *in vivo*, we have employed an animal orthotopic model of human breast cancer. MDA-MB-231 cells were inoculated into mammary fat pad of female nude mice. When the forming tumors reached the size ~20–30 mm^3^, the mice were divided into the control group (water) and the treatment group (BD 100 mg/kg of body weight/3 times/week). There were no changes in the tumor volumes for the first 2 weeks of the treatment. After that the tumors in the BD treatment group were smaller, and we detected a significant difference in the change in tumor volume over time between control and BD treatment groups (p=0.002) (Fig. 2A).

Since we used an orthotopic model of breast cancer where tumors form from the human breast cancer cells and metastasize to lungs, we evaluated the incidence of metastasis and metastasis multiplicity number in the control and BD treatment groups. In the control group we detect breast-to-lung cancer metastases in 10 of 15 animals (67%) whereas in the BD group only 3 of 15 animals (20%) developed breast to lung cancer metastasis (Fig. 2B). Therefore, the 100 mg/kg of BD significantly decreased incidence of breast-to-lung cancer metastasis by 70% (p=0.025) demonstrating preventive effect of BD against breast-to-lung cancer metastasis (Table II). Moreover, BD also significantly suppressed the amount of lung metastases from 2.8 (0.0, 48) to 0 (0.0, 14.2) (Table II).

### Effect of BD on the gene expression in tumors

**Figure 3:**
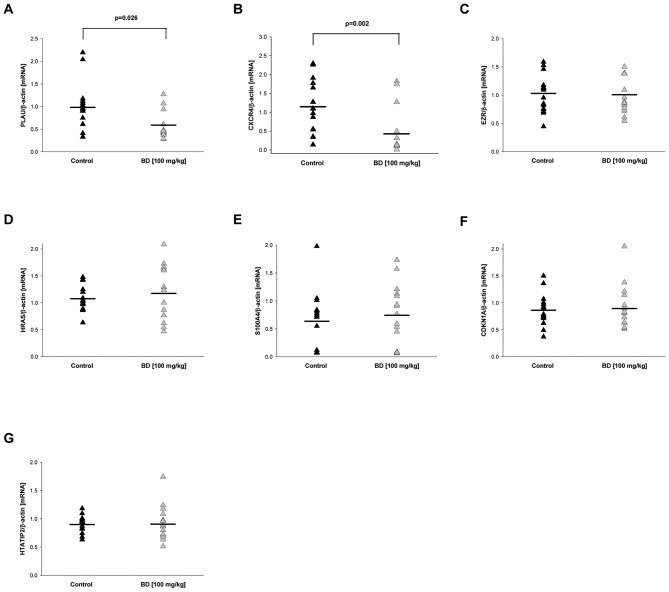
qRT-PCR in primary tumors. The mRNA expression of (A) PLAU, (B) CXCR4, (C) EZR, (D) HRAS, (E) S100A4, (F) CDKN1A, and (G) HTATIP2 in tumors from control and BD treatment (100 mg/kg) was determined by qRT-PCR as described in Materials and methods. The results are expressed as the relative expression ratios of specific mRNA to β-actin from control and BD treatment groups (n=15). The mean is indicated by the horizontal line in each group. qRT-PCR data were compared between control and BD treatment groups using Wilcoxon rank sum test.

We have recently demonstrated that BD inhibits invasive behavior and expression of uPA and CXCR4 in MDA-MB-231 cells (6). Because cell invasiveness *in vitro* reflects metastatic properties *in vivo* we hypothesized that the inhibition of breast-to-lung cancer metastasis by BD is associated with the downregulation of expression of uPA and CXCR4 in primary tumors. To evaluate the effect of BD on the expression of *PLAU* (uPA protein) and *CXCR4* genes in breast tumors, we isolated RNA and performed quantitative RT-PCR in control and BD treated mice as described in Materials and methods. In agreement with our *in vitro* study (6), BD treatment significantly downregulated expression of *PLAU* (p=0.026) and *CXCR4* (p=0.002) in breast tumors (Fig. 3). Moreover, *PLAU* and *CXCR4* expression in primary tumors was significantly increased in animals with breast-to-lung cancer metastasis (Table III). In addition, we have evaluated expression of other genes associated with breast-to-lung cancer metastasis: ezrin (*EZR*) (19), *HRAS*(20), *S100A4*(21), *CDKN1A* (protein p21) (22) and *HTATIP2* (protein TIP30) (23). However, BD treatment did not change expression of these genes in the primary tumors (Fig. 3).

## Discussion

Since cancer metastases are the major reason for the mortality of cancer patients, prevention of metastases will significantly extend life of cancer patients. Here we showed that dietary supplement BD markedly prevented breast-to-lung cancer metastases in an animal model of metastatic human breast cancer. Although we observed a modest but significant inhibition in tumor volume over time between control and BD treatment groups, this effect was not so dramatic. Nevertheless, BD treatment prevented breast-to-lung cancer metastases by 70%. The *in vivo* systemic effect of BD, after an oral application of BD, can protect against breast-to-lung cancer metastasis as we are demonstrating here.

BD is a polybotanical compound and some of its isolated components suppressed cancer metastases *in vivo*. For example, dietary administration of curcumin decreased the incidence of breast-to-lung cancer metastasis in nude mice (24). Apigenin, biologically active compound from *Scutellaria barbata* prevented hepatocyte growth factor induced lung metastasis of breast cancer cells (25). Protein-bound polysaccharide isolated from *Coriolus versicolor*, PSK (Krestin) inhibited lung metastasis in mice (26), and when combined with chemotherapy, PSK significantly prolonged survival of patients with metastatic gastric cancer (27). Isolated polysaccharides or an extract from *Phellinus linteus* suppressed pulmonary metastasis of melanoma cells in mice (28,29). In addition, triterpenoid fraction from *Ganoderma lucidum* inhibited liver metastasis (30), and *G. lucidum* in diet or i.p. injection of isolated ganoderic acid T suppressed lung metastasis, respectively (31,32). Finally, an oral administration of DIM markedly inhibited lung metastasis of murine cancer cells in mice (33). Therefore, in agreement with our *in vitro* study with BD (6) combined anti-metastatic effects of isolated components in BD could result in the prevention of breast-to-lung cancer metastasis *in vivo*.

Urokinase plasminogen activator (uPA; *PLAU* gene) is one of the major proteins involved in the invasive behavior (adhesion, migration and invasion) of cancer cells and cancer metastasis (34,35). Moreover, mice with knockout *PLAU* gene demonstrated slower growth and fewer metastases of human xenografted breast cancer cells in immunodeficient mice (36). Overexpression of the chemokine receptor *CXCR4* was originally detected in human breast cancer cells, malignant breast tumors and metastases (37). Inhibiting CXCR4 expression in breast cancer cells, using different strategies (e.g., siRNA silencing, phenotypic *CXCR4* knockout, peptide inhibitor of protein kinease-α), also suppressed breast cancer metastasis (38–40). Therefore, targeting *PLAU* and *CXCR4* expression with natural compounds should result in the suppression of breast to lung cancer metastasis. Indeed, here we show that 70% of the inhibition of breast-to-lung cancer metastases is associated with the downregulation of expression of *PLAU* and *CXCR4* in primary tumors in mice treated with BD. Moreover, the relative expression of *PLAU* and *CXCR4* in primary tumors is a predictive marker of breast-to-lung cancer metastasis because we have identified significant increase of these genes in animals with metastases.

As mentioned above, the effect of BD on the growth of primary tumors was significant albeit modest. However, we observed a dominant effect in the inhibition of breast-to-lung cancer metastases. Since BD mainly prevents metastases, combination therapy with other agents directly targeting tumor growth and/or surgical tumor resection could be considered for the alternative treatment of invasive breast cancers.

In our study we induced primary tumors and breast-to-lung cancer metastasis with MDA-MB-231 cells. These cells are characterized as basal-like/triple-negative breast cancer cells; lacking expression of estrogen receptor-α (ER), progesterone receptor (PR) and ErbB2/neu (HER2), which represent a highly aggressive breast cancer subtype, that is resistant to treatment and is associated with poor prognosis (41). Therefore, BD inhibits breast-to-lung cancer metastases in a model based on breast cancer cells which do not respond to the targeted receptor treatments (e.g., trastumazab and hormonal treatments) (42), suggesting BD as a natural dietary agent for the treatment of triple-negative therapy resistant breast cancer cells.

In conclusion, our data demonstrate that the novel dietary supplement BreastDefend (BD): i) is not toxic *in vivo* at the concentration 100 mg/kg of body weight, and ii) inhibits growth of breast tumors and breast-to-lung cancer metastases in mice. Our data suggest that BD specifically targets expression of *PLAU* and *CXCR4* in triple-negative and highly aggressive breast tumors *in vivo*. In conclusion, BD may be considered as novel polybotanical preparation for the prevention and alternative therapy of metastatic breast cancer.

## Figures and Tables

**Table I:** Levels of liver enzymes, albumin and total protein in plasma after BD treatment.

	n	Control	n	BreastDefend 100 mg/kg	n	BreastDefend 200 mg/kg	n	BreastDefend 400 mg/kg	P-value
ALT	9	83 (34, 103)	10	64 (51, 215)	10	110 (43, 470)	4	92 (63, 113)	0.488
AST	9	236 (189, 867)	9	232 (155, 396)	9	414 (149, 1261)	5	442 (197, 940)	0.483
ALP	7	157 (149, 191)	7	165 (145, 464)	6	113.5 (53, 136)^a^	6	154 (135, 207)	0.003
Albumin	9	1.6 (1.3, 1.7)	9	1.7 (1.6, 1.8)	7	1.4 (1.3, 1.4)^a^	10	1.4 (1.3, 1.5)^a^	<0.001
Total protein	6	5.3 (4.9, 5.6)	5	5.4 (5.2, 5.6)	3	4.8 (4.6, 5.2)	4	4.8 (4.6, 5.3)	0.036

Data summarized using median (min, max) and compared across groups using Kruskal-Wallis tests. Comparisons of each group with its respective control performed using Mann-Whitney U tests with significance level adjusted using the Bonferroni correction.

a A significant difference with control.

**Table II:** BD prevents breast-to-lung cancer metastasis.

Treatment	Metastasis incidence (animals with metastases, %)	Metastasis multiplicity (metastases per animal)
Control	10/15 (67)	2.8 (0.0, 48.0)
BD (100 mg/kg)	3/15 (20)^a^	0.0 (0.0, 14.2)^b^

Metastasis incidence is summarized using percentage of animals with metastases and compared between control and BD treatment groups using Fisher’s exact test;

a p=0.025. Metastasis multiplicity are summarized using median (min, max) and compared between control and BD treatment groups using Wilcoxon rank sum test;

b p=0.033.

**Table III:** *PLAU* and *CXCR4* expression based on metastasis status.

Gene	No metastases n=17	Metastases n=13
*PLAU*	0.4 (0.3, 0.9)	1.1 (0.8, 2.2)^a^
*CXCR4*	0.1 (0.0, 0.6)	1.7 (0.9, 2.3)^a^

Expression of *PLAU* and *CXCR4* was determined by qRT-PCR as in Materials and methods. The results are expressed as the relative expression ratios of specific mRNA to β-actin. Data are summarized using median (min, max) and compared between groups using Wilcoxon rank sum test;

a p<0.001.
